# Rapid Gas Hydrate Formation—Evaluation of Three Reactor Concepts and Feasibility Study

**DOI:** 10.3390/molecules26123615

**Published:** 2021-06-12

**Authors:** Florian Filarsky, Julian Wieser, Heyko Juergen Schultz

**Affiliations:** 1 Faculty of Chemistry, Chemical Engineering, University of Applied Sciences Niederrhein, Adlerstr. 32, 47798 Krefeld, Germany; florian.filarsky@gmx.de (F.F.); wieser-julian@web.de (J.W.); 2 Institute for Coatings and Surface Chemistry (ILOC), Adlerstr. 32, 47798 Krefeld, Germany

**Keywords:** gas hydrates, rapid gas hydrate formation, natural gas storage, gas separation and conditioning, spray reactor, injection, spraying, stirred-tank reactor, slurry, adapted McCabe-Thiele diagram

## Abstract

Gas hydrates show great potential with regard to various technical applications, such as gas conditioning, separation and storage. Hence, there has been an increased interest in applied gas hydrate research worldwide in recent years. This paper describes the development of an energetically promising, highly attractive rapid gas hydrate production process that enables the instantaneous conditioning and storage of gases in the form of solid hydrates, as an alternative to costly established processes, such as, for example, cryogenic demethanization. In the first step of the investigations, three different reactor concepts for rapid hydrate formation were evaluated. It could be shown that coupled spraying with stirring provided the fastest hydrate formation and highest gas uptakes in the hydrate phase. In the second step, extensive experimental series were executed, using various different gas compositions on the example of synthetic natural gas mixtures containing methane, ethane and propane. Methane is eliminated from the gas phase and stored in gas hydrates. The experiments were conducted under moderate conditions (8 bar(g), 9–14 °C), using tetrahydrofuran as a thermodynamic promoter in a stoichiometric concentration of 5.56 mole%. High storage capacities, formation rates and separation efficiencies were achieved at moderate operation conditions supported by rough economic considerations, successfully showing the feasibility of this innovative concept. An adapted McCabe-Thiele diagram was created to approximately determine the necessary theoretical separation stage numbers for high purity gas separation requirements.

## 1. Introduction

In recent decades, the importance of gas hydrate research has expanded exponentially. Gas hydrates are solid, ice-like inclusions of gases in various water structures. All of these structures consist of small dodecahedral cages and larger cages with attached hexagonal or square surfaces that vary in size and ratio [1]. About twice the amount of energy, compared to all other fossil fuels, is stored in the form of natural gas hydrates [2], which is why the exploitation via depressurization [3,4,5,6], thermal stimulation [4,5,6,7,8], carbon dioxide sequestration or geological storage [9,10,11,12,13] becomes a larger topic. Natural gas hydrates are found worldwide, especially in the form of oceanic reserves, offshore and submarine, along continental slopes, for example, off the coasts of the United States (Hydrate Ridge) and China (South China Sea), as well as in permafrost (e.g., Canada, Mackenzie Delta; Russia, Messoyakah Field) [1,2]. Natural gas hydrate deposits have even been found in inland lakes, such as Lake Baikal (Russia) [1] and the Black Sea [5]. What all natural sources have in common is that the necessary formation conditions, in particular, high pressures (offshore with correspondingly great water depths from approx. 500 m) and low temperatures close to 0 °C, must be present. Earlier but still persistent and ongoing research concerning gas hydrates was in the field of flow assurance, especially in inhibition research [14,15,16,17,18,19,20,21,22], to prevent the plugging of pipelines and related equipment by unintended hydrate formation. Although in some areas of gas hydrate research, necessary fundamentals and mechanisms are still being successfully researched and published [23,24], nowadays, the focus is shifting towards technical hydrate application possibilities in the areas of gas storage and transport [25,26], gas separation [27,28], desalination [29,30], phase transfer materials [31,32] and even electricity supply [33].

However, gas hydrate formation is an unhasty process that takes place under extreme conditions, namely high pressures and low temperatures, which, in principle, seems unfavorable for technical hydrate applications. The kinetic and thermodynamic challenges can be partially resolved by using promoters. Thermodynamic promoters, for example, tetrahydrofuran (THF), are capable of forming gas hydrates themselves at moderate conditions in terms of pressure and temperature, allowing other guests to fill the remaining empty cavities, whereby these other molecules are entrapped in the gas hydrate phase as well [34]. Furthermore, the polarity of the thermodynamic promoter affects gas diffusion via the gas-liquid interphase, which influences the selectivity and gas uptake of a gas hydrate separation process [35]. The mode of operation of kinetic promoters, essentially surfactants, is based on accelerating gas hydrate formation by reducing the surface tension, which leads to improved mass transport of gas into the liquid phase [36]. An alternative approach to improving hydrate formation is apparatus engineering measures, for instance, by using a stirred [37,38,39], packed bed [37,40,41], bubble column [42,43] or spray reactor design [44,45]; this investigation focuses on the latter approach.

Spray reactors have been the subject of previous studies. The research group around Mori et al. conducted several studies on the formation of structure H hydrates by spraying water and a promoter into various hydrocarbon atmospheres and achieved a significant rate of gas hydrate production [46,47,48]. In addition, hydrate spraying was investigated in a 15 L cell, showing successful fast methane hydrate output with a maximum uptake of more than 80% at the pilot scale [44]. An even larger test apparatus of 25 L was built and studied by Lucia et al. The investigated parameters included spraying time, reactor pressure, water load, nozzle pressure in attendance and absence of sodium dodecyl sulfate (SDS). The best conditions were found to be 80 bar, 300 ppm SDS, 3.4 °C and a 9 L water load. It was assumed that compression work is the main cost factor and the authors expected 1.47 kWh per kg of methane to be stored as energy consumption [49]. The same reactor system was also used for the formation of carbon dioxide hydrate, with optionally adding THF and SDS [50]. The accelerating effect in terms of gas hydrate formation can be explained by an increased gas–liquid interface, whereby gas is better dissolved in the liquid phase [45].

The separation of short-chain hydrocarbons from mixtures represents an important group of separations in the petrochemical industry. Cryogenic distillation is widely used in this field but has the disadvantage of considerable energy consumption [50]. In this context, studies on gas separation via gas hydrate formation with a gas mixture of methane, ethane and propane were also the subject of several investigations. Kondo et al. formed hydrates using pure water as the liquid phase and examined methane enrichment in the gas phase [51]. Short-chain hydrocarbon separation via gas hydrate formation from pure water seems appropriate only for low and high methane concentrations [52]. A study by Soltanimehr et al. showed preferential ethane entrapping in the hydrate phase and the separation efficiency enhanced with increasing pressure and decreasing temperature [53]. A methane–ethane mixture can be separated using THF because ethane and THF compete for the large cavities in sII hydrates. Moreover, a THF mole fraction of 0.06 results in only structure II [54]. Ma et al. also noted this effect [55]. At a concentration of 6 mol% of THF, ethane was remarkably enriched in the vapor phase in a pressure range of 10 to 30 bar(a) [56].

The increasing worldwide need for sustainable reduction of energy consumption in various technical applications, in particular, in gas storage, gas treatment and conditioning processes, is obvious in the context of the current environment, pollution and climate change discussions. Therefore, innovation, research, development, analysis and optimization in the field of energy processes represent an auspicious approach to a solution contribution. As it seems to be very promising to establish a hydrate-driven process as an alternative to the energy-costly cryogenic distillation for the production of pure methane, the investigation performed connects the rapid gas hydrate formation by coupled stirring, spraying and gas separation of short-chain hydrocarbons to a new, innovative process to bind and incorporate methane directly in a pumpable hydrate slurry at moderate operation conditions while using THF as a thermodynamic promoter. “Rapid” does not necessarily mean the mathematically exact description of the kinetic conditions in the first stage of these investigations but essentially the prompt and immediate, targeted formation of hydrates without significant induction times in order to make technical application processes economically attractive.

Against this background, the hydrate-driven process itself is evaluated in a feasibility study. Moreover, three different reactor concepts are compared in this work, concerning relevant process parameters, such as, for example, the hydrate formation rate and gas uptake. These three regarded reactor concepts are hydrate formation induced by the following:

1. a gas-entry stirrer,
2. spraying via a nozzle and a recycle pump,
3. a combination from spraying with additional stirring (1 + 2).

An important goal of the investigation was to create an adapted McCabe-Thiele diagram for the best identified hydrate formation system on the basis of extensive test data, with which the necessary process stages for separating the components of a gas mixture can be roughly determined according to the step-draw method. McCabe-Thiele diagrams are well known for the design of distillation and rectification columns [57,58,59]. These diagrams are equilibrium diagrams of the underlying substance mixtures, in which the substance mole fraction of a component (usually the lighter boiling component) in the liquid phase (abscissa) is plotted against the corresponding substance mole fraction of the same component in the gas phase (ordinate). This usually results in a “bulgy” curve; the bulgier it is, that is, the more it deviates from the angle bisector, the easier it is to separate a mixture into its components. The necessary theoretical separation stages in the rectification columns can be determined by means of a step-draw method [57,59]. In the context of this study, the McCabe-Thiele diagram is to be transferred analogously to the procedure for the gas purification or gas separation process via rapid gas hydrate formation in several theoretical stages. Therefore, in the adapted McCabe-Thiele trend (see Figure 3, later in the Results and Discussion section), the mole fraction of methane in the hydrate phase (ordinate) was plotted against the mole fraction of methane in the gas phase (abscissa). Since McCabe-Thiele diagrams apply to a constant pressure, a constant pressure of 8 bar(g) was used in the present experiments.

## 2. Results and Discussion

### 2.1. Reactor Designs

**Figure 1:**
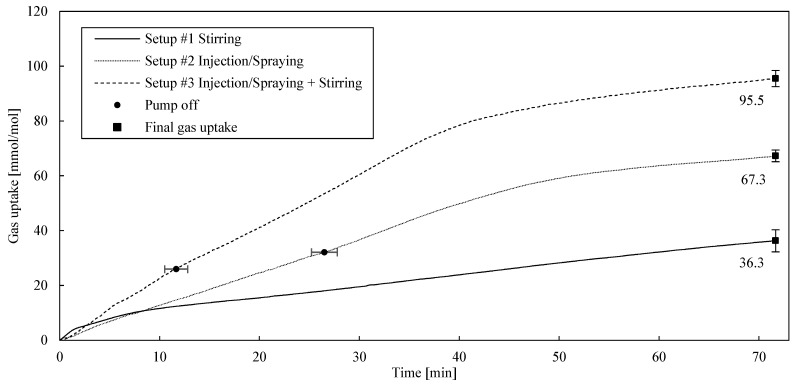
Gas uptake (mmol/mol) over time (min) for three different reactor setups.

As mentioned before, in order to prove the rapid hydrate formation in a spray reactor system, the gas uptake (mmol/mol) was recorded for three different reactor designs for classification and comparison purposes: gas-entry stirring (#1), spraying (#2) and spraying with additional stirring (#3). For these experiments, pure methane was the hydrate forming component. The results are summarized in Table 1 and the gas uptake trends can be obtained from Figure 1.

The induction times clearly show that spraying resulted in immediate nucleation after the start of the injection recycle with one exception, spraying test number three. In opposition to this, the gas-entry stirring took some time until induction occurred. The average induction time during solely stirring was 218 s.

Generally speaking, the induction times show very low absolute values between zero and six minutes. In the case of Setup #1 (stirring), these low induction times are due to the fact that the optimal operating conditions with regard to their flow conditions and turbulence were already determined in our own preliminary tests before these investigations.

Since the start of gas hydrate formation in stirring processes (Setup #1) is subject to statistical uncertainties, fluctuations are to be expected within the measurement series. Although these error values or fluctuations in Table 1 look high at first glance (218 ± 148 s), it can be clearly stated that the fluctuations in absolute terms are only between 0 and less than 2.5 min around the mean value. This is still low and in a good target range for technical applications.

The fluctuations in Setup #2 (spraying) were already significantly lower than in Setup #1 with regard to the deviation around the mean value, with 60 ± 104 s. Two of the measurements in this series of measurements showed no induction time at all, the third measurement showed only 180 s. If one also takes into account the three measurements from Setup #3, which showed no induction time twice and a negligible induction time of 2 s once, and in which stirring and also spraying took place, one can conclude from the data that spraying minimizes the induction time to extraordinarily low values. The third measurement in Setup #2 could therefore be an outlier. This hypothesis was checked in further investigations.

The experimental trends offer the conclusion that spraying with additional stirring features the highest (95.5 ± 2.9 mmol/mol) as well the fastest gas uptake, observing the experiments in the whole range, followed by the sole injection/spraying (67.3 ± 2.2 mmol/mol) and finally, the gas-entry stirring (36.3 ± 4.0 mmol/mol) as the worst process in this comparison. The accelerating effect concerning the gas hydrate formation was caused due to an enlarged gas-liquid interface, which was created by injection [45]. The gas-entry stirring design was marginally beneficial only in the few first minutes, as gas was promptly pumped into the liquid phase as long as the viscosity of the liquid was low enough. With the proceeding gas hydrate formation, the viscosity, as well as the gas-liquid phase transfer resistance driven by hydrate particles, rose and the self-priming gas-entry effect was no longer decisive.

In the case of injection, it was necessary to turn off the recycle pump after a gas uptake of approximately 30 mmol per mole of water to prevent the pump from running dry. Thus, from this pump shutdown onward, the recycling could not be operated anymore. The occurrence of such an issue was mentioned similarly by Murakami et al. in a previous study [46]. Surprisingly, the gas uptake rate did not slow down afterwards. It can be seen in Figure 1 that deactivating the pump had no effect at all on the transition time. Since a mist nozzle was used, it is supposed that fine, disperse drops of water remained as mist or fog in the vapor phase, allowing gas hydrate formation to be at a constant, unrestrained growth rate. As soon as these water drops formed gas hydrates and settled into the bulk phase, the growth rate decelerated, transitioning into a diffusion-limited process.

Moreover, in opposition to the stirring process, the injection process offered the possibility of forming gas hydrates above the liquid phase, as water drops partly condense or better set down on the reactor walls. The additional stirrer secured turbulent flow conditions in the liquid phase, also promoting gas hydrate formation. Considering all effects, the spraying process, considering coupled stirred, turbulent flow conditions, is advantageous compared to the sole gas-entry stirring and pure spraying without additional stirring.

Finally, an interesting observation can be made from Figure 1. Taking into account the final gas uptakes of the three different setups and the respective low measurement inaccuracies mentioned above, the final gas uptake of the stirring process and that of the pure injection process seem to almost add up to the value of the coupled stirring and injection process. This good agreement and obvious connection was not expected in its clarity by the authors.

### 2.2. Natural Gas Separation

All separation experiments in this section were carried out with the combined spray-injection and stirring system described above. Table 2 includes the different compositions tested by gas chromatography, measured mole fractions in the gas phase and the hydrate phase at the end of the experiment, the gas uptake in millimoles of gas per mole of water, the gas/water to hydrate conversion, the equilibrium temperature and the selectivity.

**Figure 2:**
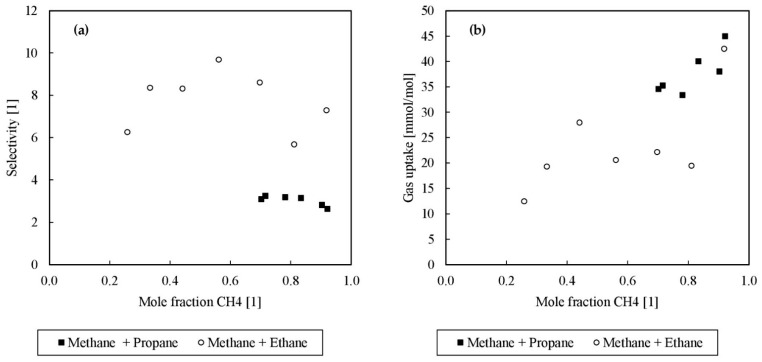
(**a**) Selectivity (ordinate) vs. initial mole fraction of methane (abscissa); (**b**) gas uptake in millimoles of gas per mole of water (ordinate) vs. initial mole fraction of methane (abscissa).

It was found that gas separation via rapid gas hydrate formation is possible in principle by the proposed combined reactor concept. As a result of THF filling the large cages in the sII hydrate structure, the small cages were occupied by methane and the other components remained mostly in the gas phase. Figure 2 illustrates the correlation between the initial mole fraction of methane and the selectivity and gas uptake.

Furthermore, Figure 2 depicts that in the comparison of ethane and propane, the selectivity of ethane is, on average, 2 times higher than that of propane, and it is thus evident that the separation of ethane from the multicomponent mixture was easier and more effective than that of propane since the higher the selectivity value, the better the separation. Propane’s worse selectivity is due to the fact that propane more strongly competes with THF for the large cages in sII. Furthermore, the gas uptake reduced with a decrease of the methane mole fraction for both composition types, whereas the methane-propane mixture showed an increased gas uptake, probably driven and amplified by the fact that not only THF hydrates incorporate methane molecules in the smaller cages but also propane hydrates do so. Both tendencies lead to the conclusion that the poorer selectivity but better gas uptake in the case of a methane-propane mixture results from propane forming sII hydrates and filling the large cages like THF. It can therefore be expected that both effects depend on the circumstance that propane is also used throughout the hydrate formation. Otherwise, the large cages of an sI hydrate structure would preferably be filled with ethane. Only at high pressures of 3000 bar(a) did Morita et al. note both cages of structure sI being occupied in pure ethane gas hydrates [60]. If the forced structure is sII, driven by the presence of THF and/or propane, ethane is massively hindered in occupying gas hydrate cages, which is a positive effect for the desired gas separation. In the investigated systems, structure sI is not in the range of possible formation conditions, so sII is preferred with the given composition and pressure and temperature conditions. Moreover, for ethane, it is hard to occupy large cages of structure sII, as these are preferentially filled by THF or propane, and the stabilizing guest-cage geometry ratios are not so favorable for ethane and the larger sII cage [1,8], otherwise, ethane would form sII instead of sI. Since the solubility of ethane in the aqueous liquid phase was rather low, and yet appreciable amounts of ethane were found in the gas phase after destabilization of the formed hydrates, it could be speculated that sI-sII mixed hydrates could be formed in the case of an increasing ethane amount, as up to a concentration of 43 mol% ethane was measured in the gas hydrate phase. This has not yet been proven but would fit as an explanation for the observations made. In order to provide the final evidence of the sI-sII coexistence, for example, RAMAN measurements would be necessary, which were done in the next steps. Nevertheless, these findings are supported by the work of Zhang et al., who found similar effects, as they formed hydrates from a methane-ethane gas mixture in the presence of 6.0 mol% THF. Under consideration of their experimental data, the selectivity was calculated to a range between 3.73 and 23.47 for 21 bar(g), and 7.16 and 21.99 for 31 bar(g) [56]. Here, the selectivity varies from 5.67 to 9.68 at 8 bar(g). Hence, an increased pressure could lead to higher selectivity and, as fewer separation stages are needed, lower investment costs at the expense of higher operation costs for an industrial process.

Throughout the experimental series, the water conversion rates moved within a range of 47.44% to 10.57%. This assumption does not take into account the occurrence of empty cavities. As the amount of methane decreased, the chance of dissolving methane and forming gas hydrate cages also decreased, which is why an initial methane mole fraction of 0.26 (Experiment No. 13) only achieved a conversion of 10.57%. This statement is supported by similar observations from Measurement Series 12, whereas Measurement Series 8 did not quite fit into the picture with regard to the conversion C (16.53%) and the selectivity S (5.67). In further investigations, the initial raw gas phase compositions were increasingly varied to allow for additional interpretations. All conversion values clarify that part of the water remained in the liquid state, hence dissolved components in the liquid phase could reduce the calculated process selectivity. Otherwise, it would be advantageous to handle a flowable gas hydrate slurry in an industrial process instead of solid-gas hydrates at the outlet. This is why a total water to hydrate conversion would not be operated in a technical application. Actually, from phase change material research with semi-clathrates, it is known that a slurry exceeding a solid clathrate phase fraction of 20 to 30 wt% ends up at an unreasonable viscosity [61]. With a lowered amount of methane, the equilibrium temperature reduces as well. Sun et al. indicated analog effects and comparable temperatures [54]. This is plausible and understandable since the stability of the gas hydrates formed also decreased in the following order: CH_4_-THF > CH_4_-Propane > CH_4_-Ethane.

Nevertheless, it is noted that with a pressure of 8 bar(g) and temperatures between 9 and 14 °C, the operating conditions were moderate. If slightly higher pressure is used, it is expected that ambient temperatures can be achieved. This will be the topic of consecutive experimental series. In comparison, typical conventional demethanizers are operated at 25 bar(g) and −83 °C [62]. This large difference in the possible operating conditions (hydrate process conditions minus cryogenic demethanization conditions: Δp = −17 bar(g); ΔT = +92 °C to +97 °C), which may not yet be the optimum, but are sufficiently meaningful in the context of this feasibility study, illustrates the great energetic potential of hydrate separation according to the process principle described, using combined spraying and stirring.

**Figure 3:**
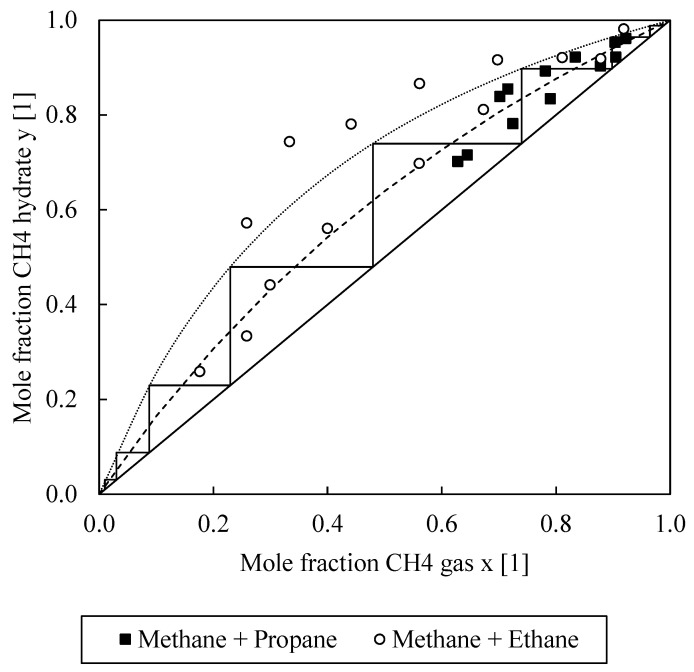
Adapted McCabe-Thiele trends in a chart where the mole fraction of methane in the hydrate phase (ordinate) is plotted against the mole fraction of methane in the gas phase (abscissa); dotted curve: equilibrium curve of the methane-ethane system; dashed curve: equilibrium curve of the methane-propane system; exemplary step-draw method pictures the necessary number of process stages for the gas separation in the methane-ethane system (8 stages). The methane-propane system is analogous but more separation stages are necessary (17 stages) and, for reasons of clarity, not shown in the diagram.

Figure 3 illustrates the mole fraction of methane in the remaining gas phase plotted against the mole fraction of methane in the solid hydrate phase. For each experiment, the ideal separation factors can be calculated, which can be used to create and construct adapted McCabe-Thiele trends, as is commonly done in the literature [57,58,59]. As done in Figure 3, it is important to evaluate the separation efficiency over the whole measurable mole fraction range to determine reliable separation factors and exclude “azeotropic points” for the construction of the adapted McCabe-Thiele diagrams. The slight scattering of the data points is due to the experimental procedure, as gas samples were covered with sampling vials for the GC measurements. In the future, it is planned to enhance the accuracy by connecting the GC system directly to the reactor system, which was not yet necessary and useful for this feasibility study. We are aware of the minor inaccuracy with this method, which is why average ideal separation factors were determined for the construction of the McCabe-Thiele trends. Therefore, these trends represent a worst-case scenario and the separation efficiency in an application case would even be better. For methane–ethane and methane-propane, average separation factor values for 8 bar(g) of $\alpha_{{\mathrm{CH}}_{4}/C_{2}H_{6}}=3.09\pm 1.59$ and $\alpha_{{\mathrm{CH}}_{4}/C_{3}H_{8}}=1.77\pm 0.47$ were obtained. To compare the results, calculations were repeated with the data of Zhang et al. In the case of methane-ethane separation, values of $\alpha_{{\mathrm{CH}}_{4}/C_{2}H_{6}}=3.30\pm 1.16$ for 21 bar(g) and $\alpha_{{\mathrm{CH}}_{4}/C_{2}H_{6}}=3.74\pm 1.40$ for 31 bar(g) were found [56]. This displays again an increased separation efficiency at higher pressures. However, in comparison with the results of Zhang et al. [56] and under considering a significantly lower working pressure (Δp = −13 bar up to Δp = −23 bar), the obtained separation factor of the CH_4_/C_2_H_6_ mixture seems to be nearly the same, which is what underlines the effectivity of the investigated process within this study.

By using the adapted McCabe-Thiele trends, it was feasible to estimate the number of stages necessary to separate a methane-ethane or methane-propane gas mixture in a hydrate formation slurry column. If, for example, a methane-ethane mixture were separated by means of gas hydrate separation, only 8 theoretical stages would be necessary to obtain both components with a purity of 99 mol%. A cryogenic distillation requires about 30 stages for the same task [62]. For reasons of clarity, Figure 3 does not also show the stage development for determining the necessary theoretical separation stages for the methane-propane separation task. However, with 17 theoretical separation stages, the result here would also be very good compared to conventional cryogenic separation. Since further measuring points are still missing in the lower part of the diagram, especially for the methane-propane system, and, as mentioned, the GC measurement is to be improved to a continuous analysis only in subsequent, further measurements, the exact number of the required separation stages is still subject to a small uncertainty. Within the scope of this feasibility study, at least a competitiveness of the separation by rapid gas hydrate formation compared to the conventional cryogenic separation can already be assumed on the basis of the curve progressions. The separation of the methane-ethane-propane mixture was also augmentedly investigated in the next project steps. In the worst case, two separate separation processes could be connected in a series on the basis of the curves obtained; in the best case, synergies could result, and possibly even side streams could be used. For exact statements on this, further experiments were carried out.

**Figure 4:**
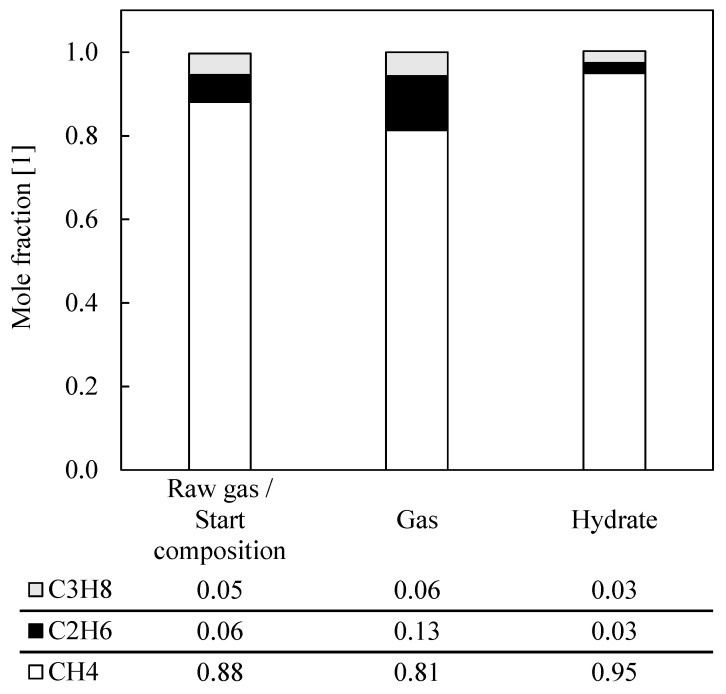
Averaged mole fractions of a synthetic natural gas as a measure of the change in composition due to gas hydrate formation; database: see Table 2, Measurement Series 14–16; left column: raw gas composition; middle column: remaining gas composition after one-stage rapid hydrate formation; right column: corresponding gas composition in hydrate phase, measured after decomposition.

After it could already be shown in the first, previous series of measurements of this investigation, as described before and shown, for example, in Figure 3, that the separation of two-component mixtures, such as methane and propane as well as methane and ethane, works convincingly and promisingly by means of a rapid gas hydrate formation process, a separation series of “natural gas” was then evaluated. Here, an exemplary, typical natural gas mixture consisting of the three representative model substances, methane, ethane and propane, was artificially generated and analyzed. The corresponding measurement data can be found in Table 2, Measurement Series 14–16. This synthetic mixture, in this case, was an example and simplification of untreated natural gas, which cannot be sold for many applications due to its lack of purity. Other trace components that are typically contained in natural gases were initially neglected in this feasibility study. The ratios of the compositions of the three components were kept largely constant. In order to be able to make an exact statement for the initial mixture, the composition was analyzed for each measurement by GC. The analytical data are summarized in Table 2, Measurement Series 14–16 and visualized in Figure 4.

The analysis resulted in a composition of the raw gas of 88 and 89 mol% methane, 7 and 6 mol% ethane and 5 mol% propane. In the following, all three measurements of this separation series were averaged. The corresponding relative total error is less than 1 mol% and can be neglected. The averaged analysis data of the separation series “natural gas” are shown graphically. The averaged raw gas contained 88 mol% methane. After the test period under consideration, the methane content in the gas phase decreased by 7 mol% and increased to 95 mol% in the hydrate phase. Corresponding to the methane decrease in the gas phase, propane increased by 1 mol%, and ethane by 7 mol%. Small proportions of ethane and propane were found in the hydrate phase, with 3 mol% each.

These results clearly show that a natural gas mixture can also be separated by means of a rapid gas hydrate formation process. It was observed that in the frame of this study, it was possible to achieve a consumer-typical gas specification with a high methane content larger than 95 mol% with a single-stage rapid gas hydrate formation process, storing methane in the form of gas hydrates, which would additionally open up the facility for transport in the form of solidified natural gas. Even though the proportion of methane in the hydrate phase appeared to be high at 95 mol%, a considerable proportion still remained in the gas phase, and this task needs to be resolved. As a result, and in order to keep the methane loss low and to further reduce impurities from ethane and propane, first enhancing the kinetics to improve the gas uptake is necessary. Second, a multi-stage process also seems to be a recommendable option, as well as further process optimization in a larger test facility. For example, an adapted, continuously operated multi-stage crystallization column is conceivable here, which would at least be procedurally similar to the proposed process. Nevertheless, it can be said that simulated natural gas separation by means of a rapid gas hydrate formation process using THF as a promoter also represents an innovative and efficient alternative for methane recovery.

To estimate the economic potential of a rapid hydrate formation process replacing the established cryogenic demethanization, a really rough and simple operation cost estimation was carried out, although this was not the key task of this study. All in all, the specific costs to operate the main cost-driving utilities in a cryogenic demethanization and a rapid gas hydrate formation process are estimated to be 2.66·10−4$mol and 1.33·10−4$mol, respectively. Knowing that the cost estimation is only a by-product of this work and a rough estimate of the costs of both processes (conventional cryogenic demethanization and rapid gas hydrate formation), the fact that rapid hydrate formation is about half as expensive in operation costs is so attractive that regardless of possible higher installation costs, a fast break-even point and huge economical potential can be assumed. This finding motivates the continuation of this research and makes clear that the studies presented here should be intensified and expanded in the future.

Although the separation efficiency was quite promising, some challenges occurred at this point. First, the water to hydrate conversion was still quite low regarding an application for the production of solidified natural gas. Therefore, a longer residence time and further improvement of the reactor design are necessary to achieve an even faster and more complete gas hydrate formation. In addition, methane is the dominating component of natural gas. Hence, if methane is not the desired component, which is stored in the form of gas hydrates, it would be more practical to selectively remove ethane and propane from the gas stream, as the technical handling of a much smaller amount of a hydrate-forming mixture would then be required. This approach was already the subject of former works. In this study, no THF was used, only water. Uchida et al. observed a two-step gas hydrate crystallization while using a gas mixture of methane and propane [63]. First, a mixed methane-propane sII gas hydrate was formed, resulting in a methane-rich gas phase. In the case of methane, with partial pressure higher than the dissociation pressure of pure methane hydrates at a specified temperature, sI methane hydrates began to crystallize after some time. This enabled the possibility of separating methane from propane if the partial pressure of methane was lower than the equilibrium pressure after the first crystallization step with propane involved. This two-step mechanism was confirmed for methane-propane gas hydrates as well as methane-ethane gas hydrates [64]. Regarding an ethane-propane gas mixture, Al-Otaibi et al. discovered that there is a dependency on the gas composition, that is, which gas hydrate structure, either sI or sII, is formed [65]. There was a shift from structure sII to sI at an ethane mole fraction of 0.73. While using an equimolar gas composition, structure sII was formed with no separation effect. The composition of the gas phase remained unchanged during the whole experimental procedure. If all three gases were present, all three gas components could be found in the large gas hydrate cages, whereby the small cages were about to be filled with methane by 90%, resulting in a methane-rich gas phase [66]. However, a tremendous problem can occur during the separation of a model natural gas containing methane, ethane and propane, as Ando et al. found [67]. While using an SDS solution for the gas hydrate formation, a shift in the gas hydrate structure from structure sII to sI was observed, presumably. First, this resulted in the known enrichment of methane in the gas phase during the formation of structure sII, followed by a decreasing methane mole fraction in the gas phase as the transition occurred, which released either the ethane or the propane.

In summary, during the separation of the model natural gas and in the absence of the thermodynamic promoter THF, ethane and propane occupied the large cages and methane occupied the small cages of a structure sII gas hydrate, resulting in a methane-rich gas phase. This would be the better option to obtain pure methane, as ethane and propane represent impurities of low molar fractions. However, the separation efficiency depends significantly on the operation conditions, which can even result in a reversed process. Therefore, both gas hydrate forming liquids, water and water-THF, offer advantages and disadvantages and additional experiments are necessary in order to improve the selectivity and the targeted and controllable entrapment of only ethane and propane in the gas hydrate phase. The experiments carried out within the framework of this study are an important and promising contribution that can be used as a valuable starting point for follow-up experiments to develop an economically attractive, innovative, energy-saving and sustainable process.

## 3. Materials and Methods

The main focus of the study was to:

- Evaluate the optimal process for rapid gas hydrate formation under consideration of three different reactor concepts;
- Examine the feasibility of the rapid hydrate formation from gas mixtures for the application in (natural) gas storage and conditioning.

**Figure 5:**
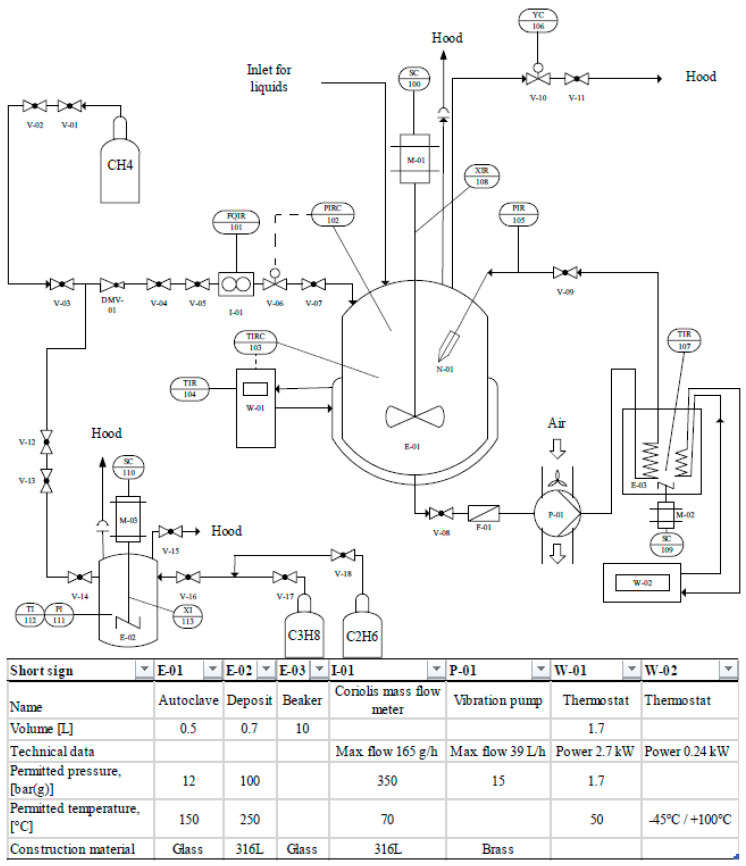
Simplified P&ID of the experimental setup.

Figure 5 shows the experimental setup. For reasons of clarity, only the most important items of equipment are listed in the equipment bar.

**Figure 6:**
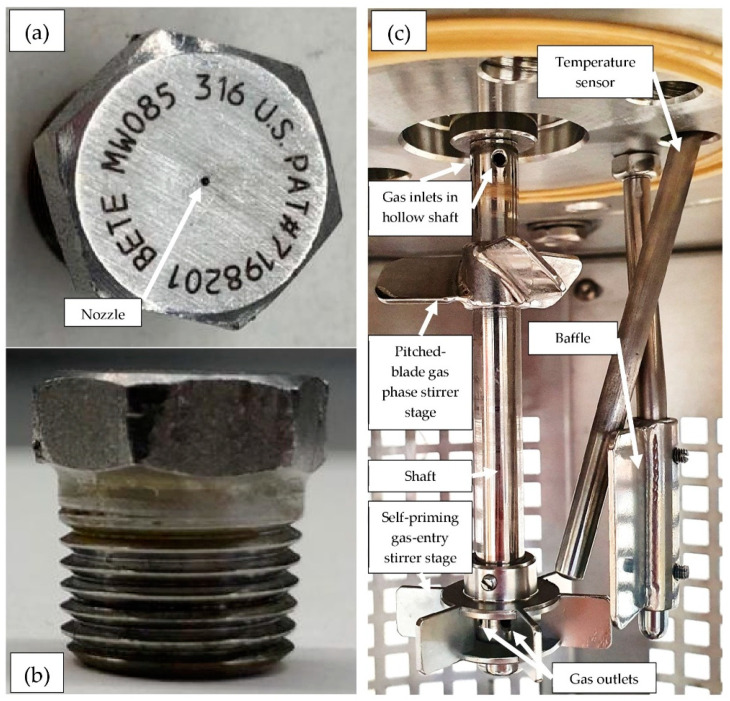
Reactor internals: (**a**) Micro Whirl MW85 injector nozzle, by Bete, Greenfield, MA, USA, top view, injecting a cone-shaped mist with a spray angle up to 70° and an approximate flow rate of 0.04 L/min; (**b**) Bete Micro Whirl MW85 injector nozzle, side view; (**c**) stirring equipment, clockwise direction of rotation.

The experiments were conducted in a 0.5 L reactor (inner diameter D = 0.082 m) manufactured by Büchi AG, Uster, Switzerland ”ecoclave” Type 1 (E-01, see Figure 5). This 0.5 L reactor is available in our laboratory in two versions: in the glass version with limitations at about 12 bar(g) and 200 °C, and as a stainless steel version, which is approved for up to 60 bar(g) and 250 °C. The reactor head for both types is the same and provided with a stirrer motor type “RZR2102” by Heidolph Instruments GmbH & Co. KG, Schwabach, Germany (M-01), a “mini CORI-FLOW” Coriolis mass flow system manufactured by Bronkhorst Deutschland Nord GmbH, Kamen, Germany (I-01) and a “Presto A40” thermostat by Julabo GmbH, Seelbach, Germany (W-01). An optional recycle pump for the spray injection was installed, comprising a Micro Whirl MW85 nozzle by Bete, Greenfield, MA, USA (X-01), injecting a cone-shaped mist with a spray angle up to 70° and an approximate flow rate of 0.04 L/min (see Figure 6a,b), an electromagnetic vibration recycle pump Ulka EX5 by Scintilla pumps Ltd., Wallingford, UK (P-01) and a recycle cooling coil (E-03).

In order to achieve a rapid gas hydrate formation, the experimental series was conducted with a two-stage, self-priming, gas-entry stirring system, as can be seen in Figure 6c, comprising a pitched-blade stirrer stage installed in the vapor phase and the additional self-priming, paddle-blade stirrer stage immersed in the liquid phase. In previous experiments of our own, it was found that this kind of mixing setup is advantageous with regard to the speed of the hydrate formation. A self-priming, gas-entry stirring system means, in this context, that the agitator shaft was hollow and gas was sucked in from the gas phase via openings in the agitator shaft from a minimum rotational frequency, which could be estimated in a simplified manner using the Bernoulli equation, and the gas was finely dispersed as bubbles in the liquid phase via further openings in the agitator element. The 6 paddle blades distributed the escaping gas bubbles radially in the liquid and the high turbulence and corresponding vortices in the liquid phase broke up the bubbles into even smaller aggregates. This increased the phase interface between the liquid and gas phases necessary for hydrate formation.

The stirrer was operated at a rotation frequency of 900 rpm (15 rps), leading to a stirrer Reynolds number of Re_S_ ≈ 24,999. The advantage of using the dimensionless stirrer Reynolds number as a measure of the flow regime and turbulence in the system instead of using exclusively rotational frequencies is that dimensionless numbers are suitable for scale transfer. Keeping the Re_S_ constant is a much better comparison criterion between different apparatuses than naming rotational frequencies. From the point of view of stirring technology, the formation of gas hydrates by means of often-used stirrer bars (“stirring fishes”) has hardly anything to do with stirring devices used on a large scale, which is why the gas hydrate experiments in this investigation were carried out with a proper, realistic, two-stage stirring system for a more reliable transfer of scale. The value of Re_S_ ≈ 24,999 represents a turbulent mixing regime. Equation (1) shows that the stirrer Reynolds number Re_S_ represents the ratio of the inertial force to the internal frictional force of the stirred medium.

(1) ReS=f·d2·ρliqηliq=15s−1·0.052m2·999.94kgm31.5·10−3 Pa·s≈24,999

The second stirring element (pitched-blade stirrer) additionally mounted on the stirrer shaft in the gas phase also imprinted an axial flow profile on the gas phase in the direction of the phase interface, whereby the mass transport necessary for hydrate formation could be significantly improved.

The temperature and pressure were controlled and measured with a process control system, “deltaV”, delivered by Emerson Electric Co., Saint Louis, MO, USA. The pressure sensor was a SITRANS P200 (Siemens AG, Munich, Germany), with an accuracy of ± 0.04 bar, and the temperature sensor was a Juchheim PT100 (JUMO GmbH & Co. KG, Fulda, Germany) with an accuracy of ±0.15 + 0.002 · ϑ K.

A Sartorius “Extend ED3202S-CW” (Sartorius Lab Instruments GmbH & Co. KG, Goettingen, Germany) was used for weighing and, in the case of necessary gas analyses in part two of the study, these were performed via an Agilent type “6890 Series” gas chromatography system equipped with an Agilent J&W HP-PLOT U column (both by Agilent Technologies, Santa Clara, CA, USA), suitable for analyzing the specific gas atmospheres. The GC measurements had an accuracy of ≤0.01 mol%. Gas mixtures of pure methane, ethane and propane with a purity of ≥99.5%, delivered from Messer Industriegase GmbH, Bad Soden, Germany, were prepared and stored in a 0.7 L reactor (E-02) by Amar Equipments Pvt. Ltd., Mumbai, India. Sigma Aldrich, St. Louis, MO, USA provided tetrahydrofuran with a purity of ≥99.9%.

In the subsequent series of experiments, THF was used as a thermodynamic promoter at the stoichiometric concentration of 5.56 mol% to ensure the formation of structure sII hydrates and the incorporation of THF in all large cages and not with gas molecules. The selected pressure and temperature conditions (8 bar(g), 9–14 °C) ensured that mixed hydrates of sII and sI did not occur, which could have complicated the investigation and possibly reduced the selectivity and separation efficiency during the natural gas separation experiments, which was undesirable.

Due to the high volatility of THF, experimental problems in the measurement of the gas composition were to be expected, but these turned out to be negligible, as traces of THF were not detected in the GC measurements. For each gas separation experiment the mole fractions in the initial mixture, gas phase and hydrate phase, the equilibrium temperature T_eq_ (°C), the gas uptake (mmol/mol), the selectivity S (1) and the conversion C (%) were determined. The selectivity S was calculated to determine the separation efficiency (Equation (2)).

(2) $$S=\frac{x_{CH_{4}}^{Hyd}*x_{C_{2}H_{6}/C_{3}H_{8}}^{Gas}}{x_{CH_{4}}^{Gas}*x_{C_{2}H_{6}/C_{3}H_{8}}^{Hyd}}$$

Equation (2) describes the ratio between the mole fractions of methane $\left(x_{CH_{4}}^{Hyd}\right)$ and ethane or propane $\left(x_{C_{2}H_{6}/C_{3}H_{8}}^{Hyd}\right)$ in the hydrate and in the gas phase $\left(x_{CH_{4}}^{Gas},\;x_{C_{2}H_{6}/C_{3}H_{8}}^{Gas}\right)$. The higher the value, the better the separation, as a complete separation process would lead to an infinite value.

The conversion was calculated conservatively, assuming that using 5.56 mol% THF secured a filling of all large cages in structure II. As the unit cell can be described as $16\cdot 5^{12}*8\cdot 5^{12}6^{4}*136\cdot H_{2}O$, the ideal maximum methane gas uptake in mmol/mol would be $\frac{16\;\mathrm{mol}}{136\;\mathrm{mol}}\approx 118\frac{\mathrm{mmol}}{\mathrm{mol}}$. The measured gas uptake in g divided by the used amount of water in g can be converted to the unit mmol/mol (see Equation (3)).

(3) $$Gas\;Uptake=\frac{m_{Gas}}{m_{Water}}=\frac{\sum_{i=1}^{j}n_{Gas,i}\cdot M_{Gas,i}}{n_{Water}\cdot M_{Water}}\cdot 1000\;\left[\frac{\mathrm{mmol}}{\mathrm{mol}}\right]$$

Therefore, the conversion can be calculated. In Equation (4) $n_{Gas}$ and $n_{Water}$ typify the molar amount of consumed gas and used water respectively:

(4) $$C=\frac{n_{Gas}}{n_{Water}*118\frac{\mathrm{mmol}}{\mathrm{mol}}}*100\;\left[\%\right]$$

Please note that this is an ideal estimation; the conversion could be higher as empty cages and real hydrate numbers were left unconsidered. Nevertheless, this led to appropriate results for the discussion.

In order to be able to evaluate the three different reactor configurations and make a reliable statement about the optimum process variant, in the first collection of data, an experimental series was performed to confirm the rapid gas hydrate formation of the proposed reactor types and processes. Following this, as a benchmark test, three different reactor setups were tested considering their performance to form mixed methane-THF hydrates. The first setup worked without using the spray nozzle but had a self-priming gas-entry stirrer, comprising a pitched-blade stirrer stage in the vapor phase and a self-priming paddle-blade stage in the liquid phase (see Figure 6c). The second design only comprised the injection via the recycle pump and nozzle (see Figure 6a,b) without stirring, whereas the third one covered the same experimental setup as used in the separation experiments described in the second part of this chapter, namely the injection via a recycle pump with additional stirring of the liquid phase. The experimental procedure was conducted in the following order:

For the injection experiments (Setups #2 and #3), 370 mL of the gas hydrate-forming mixture comprising distilled water and 5.56 mol% THF was filled in the autoclave. In this context, consideration must be given to the dead volume of the circulation line. The reaction mixture that was outside the reactor within the recycle pipe between the reactor outlet and the spray nozzle did not take part in the reaction and was not converted into hydrate. This liquid dead volume was approx. 120 mL and filled the recycle line as a hold-up directly at the start of operation, when the recycle pump was switched on. For all test series in which a nozzle process is considered (#2 and #3), the dead volume must therefore be added to the reaction volume of the reactor vessel, which results in a total volume of 370 mL. The “stirring” series of experiments (Setup #1) was carried out with the outlet closed, which is why no dead volume and no recycling have to be taken into account here. Therefore, in the case of the comparative stirring experiments, only 250 mL of the gas hydrate-forming liquid was used to guarantee the same reaction volume and liquid level (≈4.7 cm) in all setups. This led to identical and comparable operating conditions with regard to the liquid phase in all process variants considered, making it possible to contrast and transfer the different measurement series. After filling the reactor setups, the gas hydrate reactor was purged to a pressure of 5 bar(g) with the same gas mixture as used for the experiments. After purging three times, the autoclave was cooled down to 6 °C and the pressure was set to 8 bar(g). Dependent on the experimental series, either the pump for the recycle injection via the nozzle and/or the stirrer (900 rpm, stirrer Reynolds number Re_S_ ≈ 24,999) was started. Considering the injection experiments, the injection pressure difference via the nozzle was 11 bar and the temperature of the external cooling coil was held constantly at 6 °C. The experiments were stopped after 80 min and were repeated three times each. Finally, comparisons were drawn between the experimental results of the gas-entry stirring, injection and injection plus stirring. No identifiable flow restrictions were observed during the operation of the nozzle, except for a lack of liquid level after a certain circuit operating time, as the liquid phase has been increasingly incorporated into the solid hydrate phase. Therefore, to save the recycle pump from running dry, it had to be turned off after a certain time (see Figure 1) at a gas uptake of approximately 30 mmol/mol.

After successfully and very promisingly proving the feasibility of the coupled stirring and spray reactor design, in the second part of the study, the test procedures for the gas separation and conditioning experiments had the following procedure. The deposition autoclave (E-02, see Figure 5) was purged and filled with the appropriate gas mixture. The reactor for the gas hydrate formation (E-01) was filled with 370 mL of the gas hydrate-forming mixture comprising distilled water and 5.56 mol% THF. Then, the gas hydrate-forming reactor was purged up to a pressure of 5 bar(g) three times with the experimental gas mixture. After purging, a pressure of 8 bar(g) was set, the pump (P-01) was started for circuit injection via the nozzle (X-01) and/or the stirrer (M-01) (900 rpm, stirrer Reynolds number Re_S_ ≈ 24,999) and the reactor was cooled until gas hydrate induction. The temperature of the external cooling coil was kept at 6 °C, as preliminary tests have shown that this allows the temperature of the circulating flow outside the reactor to be kept constant, thus preventing it from heating up due to higher temperatures of the surrounding laboratory. Since temperature control was slow, a slight heat accumulation was recognized during the course of the hydrate formation, finally leading to the hydrate formation/decomposition equilibrium temperature at the adjusted pressure of 8 bar(g), as gas hydrate formation and decomposition are in equilibrium at this state. Now, the thermostat (W-01) temperature was fixed to the corresponding equilibrium temperature. This series of experiments were stopped after 50 min, whereupon gas samples were taken for analysis purposes. After this, the pressure was quickly lowered to ambient conditions. After this decompression, all valves were closed again, and by raising the temperature to 20 °C, the remaining gas hydrates were decomposed. The reactor pressure raised again because of the decomposing gas hydrates, and the released gas phase had almost the composition of the gas molecules in the former solid gas hydrates, which was determined by analyzing another gas sample. In addition, the original gas composition was measured by analyzing a gas sample from the deposit autoclave. The gas absorbed during the experiment and remaining in the residual liquid cannot be taken into account in this experimental procedure. Hence, the results represent a kind of a worst-case scenario, as the measured separation efficiencies are influenced by possible minor contamination of the desorbed components from the liquid phase. The residual gas at atmospheric pressure after the first autoclave venting was mathematically taken into account.

## 4. Conclusions

This advanced feasibility study on natural gas separation and conditioning using rapid gas hydrate formation serves as a proof-of-concept and demonstrates the distinct potential of this process in the presented application case of low-hydrocarbon processing.

In the process development step of this work, the combination of a spraying and a stirring process was successfully identified and described as the most promising variant by comparing three different technical hydrate formation methods.

Based on these findings, an application method could be demonstrated in the evaluation step of this feasibility study, which provided a prompt, immediate, fast gas hydrate formation with negligible induction times at moderate pressure (8 bar(g)) and temperature (9 to 14 °C) conditions and, due to the utilization of the thermodynamic promoter tetrahydrofuran (THF), methane was stored in the form of a flowable hydrate slurry, which could be easily converted into solidified natural gas.

Additionally, the purpose of constructing an adapted McCabe-Thiele diagram was successfully implemented to estimate the necessary number of stages of a multi-stage process to separate methane from ethane and propane by rapid hydrate formation. In order to achieve this, extensive tests with different, varying gas compositions were carried out. It was determined that the selectivity towards ethane is higher than towards propane because ethane is not or rarely included in a structure sII hydrate when using THF. As the amount of ethane increases, a mixed sI–sII hydrate is likely to form, reducing the separation efficiency. THF and propane compete with higher intensity for the large cages of structure sII, resulting in poorer selectivity but increased gas uptake. However, the separation efficiencies for both ethane and propane are very promising in regard to mixing with methane.

The next steps for this project are (I) the clarification of the formed hydrate structures by RAMAN measurements; (II) connecting the GC system directly to the reactor system that leads to better McCabe-Thiele trends due to enhanced sampling accuracy; (III) expansion of the database in other compositions of the raw gas to refine the McCabe-Thiele trends; (IV) improvement of water to hydrate conversion by adapting the reactor design; (V) investigation of slightly higher pressures to reach ambient temperatures; (VI) further process optimization in a larger test facility (adapted, continuously multi-stage crystallization column); finally, (VII) cooperation with industrial partners in the concerned sector to implement the process in a pilot plant.

As a by-product of this work, a rough estimate of the costs for both processes, conventional cryogenic demethanization and the novel rapid hydrate formation, indicates that the hydrate-driven process is about half as expensive in operation costs and, therefore, is economically very attractive. Regardless of possible higher installation costs, a fast break-even point and a huge economical potential can be assumed.

Next to this, considering the necessity of energetic, ecological and sustainable improvement to large-scale processes, a great advantage is to be expected when using the proposed process whose feasibility has been successfully proven. Energy and material consumption are lower, and the ecological footprint is significantly better. Further development of the rapid gas hydrate formation technology for fast natural gas storage and conditioning seems to be worth it, both from an economic and ecological point of view.

## Figures and Tables

**Table 1:** Experimental results for three different reactor setups.

Exp.	No.	Induction Time (s)	Ø Induction Time (s)	Final Gas Uptake (mmol/mol)	Ø Final Gas Uptake (mmol/mol)	Ø C (%)
Setup #1 Stirring	1	287	218 ± 148	33.3	36.3 ± 4.0	30.8 ± 3.4
	2	48		34.7		
	3	319		40.9		
Setup #2 Spraying	1	0	60 ± 104	69.7	67.3 ± 2.2	57.0 ± 1.8
	2	0		66.6		
	3	180		65.5		
Setup #3 Spraying + Stirring	1	0	1 ± 1	93.9	95.5 ± 2.9	80.9 ± 2.5
	2	2		98.9		
	3	0		93.7		

**Table 2:** Experimental results, equilibrium pressure 8 bar(g).

Nr.	Initial Composition			Gas Phase			Hydrate Phase			Gas Uptake	C	T_eq_	S
	(mol%)			(mol%)			(mol%)			(mmol/mol)	(%)	(°C)	(1)
	CH_4_	C_2_H_6_	C_3_H_8_	CH_4_	C_2_H_6_	C_3_H_8_	CH_4_	C_2_H_6_	C_3_H_8_				
1	0.92		0.08	0.90		0.10	0.96		0.04	44.97	38.22	14.24	2.63
2	0.90		0.10	0.88		0.12	0.95		0.05	38.01	32.31	14.15	2.81
3	0.83		0.17	0.79		0.21	0.92		0.08	40.04	34.04	13.63	3.14
4	0.78		0.22	0.72		0.28	0.89		0.11	33.34	28.34	14.11	3.18
5	0.72		0.28	0.64		0.36	0.85		0.15	35.26	29.97	13.74	3.24
6	0.70		0.30	0.63		0.37	0.84		0.16	34.58	29.39	13.45	3.08
7	0.92	0.08		0.88	0.12		0.98	0.02		42.50	36.13	13.45	7.29
8	0.81	0.19		0.67	0.33		0.92	0.08		19.44	16.53	13.20	5.67
9	0.70	0.30		0.56	0.44		0.92	0.08		22.17	18.84	12.80	8.60
10	0.56	0.44		0.40	0.60		0.87	0.13		20.58	17.49	10.91	9.68
11	0.44	0.56		0.30	0.70		0.78	0.22		27.96	23.77	10.29	8.31
12	0.33	0.67		0.26	0.74		0.74	0.26		19.28	16.39	9.78	8.35
13	0.26	0.74		0.18	0.82		0.57	0.43		12.44	10.57	8.74	6.25
14	0.88	0.07	0.05	0.84	0.11	0.05	0.96	0.02	0.02	42.92	36.49	13.32	5.13
15	0.88	0.07	0.05	0.81	0.13	0.06	0.94	0.03	0.03	40.43	34.37	13.45	3.76
16	0.89	0.06	0.05	0.81	0.13	0.06	0.94	0.03	0.03	55.81	47.44	13.27	3.73

## Data Availability

The data presented in this study are available on request from the corresponding author.

## Abbreviations

CH_4_	methane
C_2_H_6_	ethane
C_3_H_8_	propane
eq	equilibrium
GC	gas chromatography
i	gas component in simulated natural gas
j	summation index
rpm	rounds per minute
rps	rounds per second
sI	structure I hydrate
sII	structure II hydrate
S	Stirrer
SDS	sodium dodecyl sulfate
THF	tetrahydrofuran
Formula symbols:	
C	conversion (%)
d	diameter of stirrer (m)
D	inner diameter of reactor (m)
f	stirrer frequency (s^−1^)
m	mass (g)
M	molar mass (g/mol)
n	molar amount (mol)
Re_S_	stirrer Reynolds number (1)
S	selectivity (1)
x	mole fraction (1)
α	average separation factor (1)
ρ	density of hydrate forming liquid phase (kg/m^3^)
η	dynamic viscosity of hydrate forming liquid phase (Pa s)
Δp	pressure difference (bar)
ΔT	temperature difference (°C)
